# THE BLUES AND OLDER MINORITY MUSICIANS: MORE THAN JUST MUSIC XXXI

**DOI:** 10.1093/geroni/igae098.2242

**Published:** 2024-12-31

**Authors:** John Migliaccio

**Affiliations:** Maturity Mark Services Co., LLC, White Plains, New York, United States

## Abstract

The Blues and Older Minority Musicians: More Than Just Music has been a session at GSA for 31 years straight.

